# Malignant adenohypophysis spindle cell oncocytoma with repeating recurrences and a high Ki-67 index

**DOI:** 10.1097/MD.0000000000005657

**Published:** 2017-01-27

**Authors:** Xiangyi Kong, Dongmei Li, Yanguo Kong, Dingrong Zhong

**Affiliations:** a Department of Neurosurgery, Peking Union Medical College Hospital, Chinese Academy of Medical Sciences, Beijing, China; b Department of Anesthesia, Critical Care and Pain Medicine, Massachusetts General Hospital, Harvard Medical School, Harvard University, Boston, MA; c Department of Pathology, Peking Union Medical College Hospital, Chinese Academy of Medical Sciences, Beijing, China.

**Keywords:** adenohypophysis spindle cell oncocytoma, Ki-67 index, malignant, recurrent

## Abstract

Adenohypophysis spindle cell oncocytoma (ASCO) is a rare tumor recently reported by Roncaroli et al in 2002. This tumor is considered a grade I tumor by the World Health Organization.

We report a rare case of malignant ASCO with repeating recurrences and a high Ki-67 index—a challenging diagnosis guided by clinical presentations, radiological signs, and postoperative pathological tests.

We represent a 30-year-old man who had suffered from headaches, diplopia, and impaired visual field and acuity. His magnetic resonance imaging revealed an abnormal sellar mass and was originally misdiagnosed as a pituitary macroadenoma. We present detailed analysis of the patient's disease course and review relevant literature.

When surgically treated, the specimen revealed a typical histopathology pattern of ASCO. The tumor recurred for several times and the patient underwent 3 surgeries and 1 γ-knife treatment, which was accompanied by a continuously increasing Ki-67 index.

This is the first reported case of malignant ASCO (WHO III–IV grade). Despite its rarity, ASCO should be considered in the differential diagnosis of sellar lesions that mimic pituitary adenomas.

## 1 Introduction

Adenohypophysis spindle cell oncocytoma (ASCO) is a recently described entity that was recognized by the 2007 WHO Classification of Brain Tumors and considered a WHO grade I tumor.^[1]^ It was initially described by Roncaroli et al^[2]^ in 2002, defined as a spindled-to-epithelioid, oncocytic, nonendocrine neoplasm of the anterior hypophysis that manifests in adults and follows a benign clinical course. The pathogenesis and prognosis of ASCO remain uncertain and need to be documented more thoroughly in the literature. Up to now, only a small number of ASCO cases have been reported in the literature. Partly because the incidence of ASCO is so low, with respect to its radiologic characteristics and recommended treatment of choice, the related literatures are very few. Usually, ASCOs could be easily misdiagnosed as nonfunctional pituitary adenomas due to their similar radiologic characteristics and symptoms and signs.^[3]^ Since the blood supply to ASCO is usually much richer than that to the pituitary adenoma, the operation for ASCO is thus more difficult.^[4]^ We are aimed at reporting a new case of ASCO with repeating recurrences and a high ki-67 proliferation index of 45% (WHO III–IV), which is very different from previously reported ones in malignancy issue. Related literatures are also reviewed in our study.

## 2 Case presentation

A 30-year-old man came to Peking Union Medical College Hospital (PUMCH) with headaches, fatigue, diplopia, and impaired visual field and acuity for 6 months which had worsened since the previous 2 weeks. He denied polydipsia, polyuria, sexual hypoactivity, or any symptoms of unconsciousness, epilepsy, convulsion, and cognitive disorders. Physical examinations revealed that his right visual acuity was 0.5 and the left was 0.4. Goldmann perimetry revealed a bitemporal hemianopia. He was found to have ptosis of the right eyelid. The right pupillary reaction to light was absent. Other neurological examination results were normal. His past history was negative for head trauma. His social and family history and his system review were negative.

**Figure 1:**
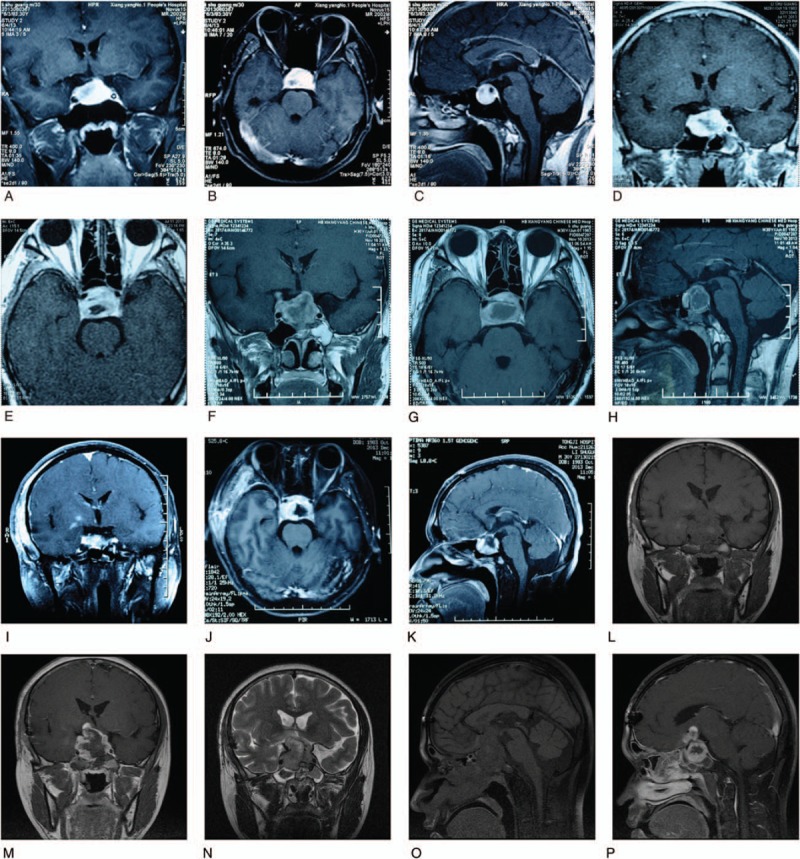
MRI for the abnormalities in the sellar region at different time points. A–C, Before the first surgery. A, Coronal-enhanced T1WI. B, Axial-enhanced T1WI. C, Sagittal-enhanced T1WI. D–E, Before γ-knife treatment. D, Coronal-enhanced T1WI. E, Axial T1WI. F–H, Before the secondary surgery. F, Coronal-enhanced T1WI. G, Axial T1WI. H, Sagittal-enhanced T1WI. I–K, After the secondary surgery. I, Coronal-enhanced T1WI. J, Axial-enhanced T1WI. K, Sagittal-enhanced T1WI. L–P, Before the third surgery. L, Coronal T1WI. M, Coronal-enhanced T1WI. N, Coronal T2WI. O, Sagittal T1WI. P, Sagittal-enhanced T1WI. MRI = magnetic resonance imaging.

The magnetic resonance imaging (MRI) demonstrated an abnormal mixed signal mass with suprasellar, parasellar, and suprasellar invasiveness in the sellar area (Fig. 1A–C). The lesion was about 2.8 × 1.9 × 1.9 cm, inside which was some spotty necrosis. A dynamic contrast-enhanced scan showed heterogeneous enhancement. Relatively normal pituitary tissue with normal enhancement could be seen near the inferior lesion margin, but was squashed. The optic chiasma was mildly compressed but the basic shape was generally normal. The bilateral internal carotid arteries were also partly wrapped. Laboratory tests used to explore pituitary disorders showed normal levels of pituitary hormones, including prolactin (N < 20 μg/L), luteinizing hormone (LH) (N > 10 IU/L), follicle-stimulating hormone (FSH) (N > 20 IU/L), thyrotropin, and corticotropin. The diagnosis of nonfunctioning pituitary macroadenoma was suspected.

**Figure 2:**
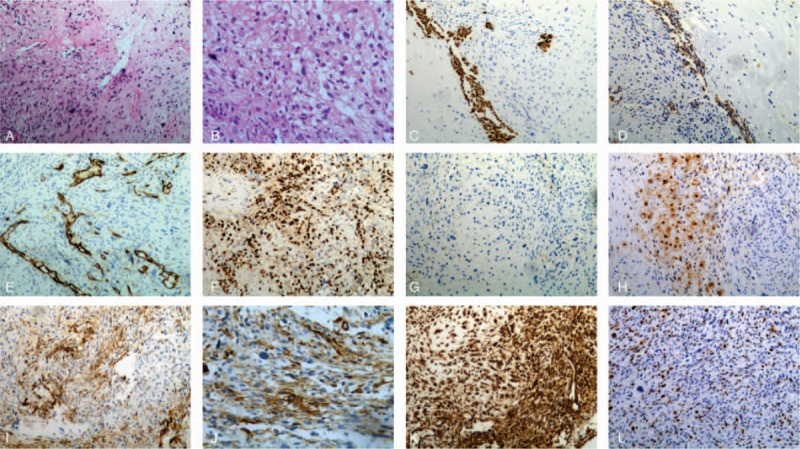
Histopathological and immunohistochemical examination images for the surgical specimen. A–K, Specimen after the first surgery. A, H & E, ×10. B, H & E, ×20. C, AE-1/AE-3, ×10. D, AS-1, ×10. E, CD34, ×10. F, CD68, ×10. G, Desmin, ×10. H, S-100 protein, ×10. I, SMA, ×10. J, SMA, ×20. K, Vimentin, ×10. L, Specimen after the third surgery, Ki-67 proliferation index 45%, ×10. H & E = hematoxylin and eosin, SMA = smooth muscle actin.

Via a trans-nasal-sphenoidal approach, a surgical exploration was performed. After drilling the sellar floor and opening the dura, a firm, tough, wheaten mass was found. As its consistency was too elastic and hypervascular to be easily cut by a surgical blade, and it adhered so tightly to the cavernous sinus and internal carotid artery, only subtotal resection was ultimately achieved. Repair of the sellar defect was done with autologous fat and fascia lata. The immediate postoperative sellar MRI was not performed. In a surprise twist, postsurgical hematoxylin and eosin (H & E) stained sections showed that the lesion contains epithelioid and spindled cells with eosinophilic cytoplasm arranged in sheets and nests. Mild-to-moderate nuclear atypia could also be observed. On immunohistochemical evaluation, the tumor cells were positive for Vimentin, CD68, CD34, Nestin, GFAP, Desmin, SMA, AE1/AE3, and S-100 protein, but were negative for NSE, Synuclein, NeuN, EMA, pituitary hormones (LH, FSH, ACTH, TSH, growth hormone, and prolactin), synaptophysin and chromogranin. Ki-67 proliferation index was 6% (Fig. 2A–K). The pathological test supported the diagnosis of adenohypophysis spindle-cell oncocytoma. The postsurgical course was uneventful and his clinical symptoms of headache and diplopia were markedly improved.

However, 1 month after the surgery, the patient's vision declined sharply and meanwhile he complained of severe ophthalmodynia of the right eye. The sellar MRI revealed that the tumor recurred (Fig. 1D and E) and the patient underwent a γ-knife treatment. But the symptoms were not relieved significantly. Three months later, the sellar MRI showed the lesion was approximately 2.8 × 2.2 × 3.1 cm with equal T1 signal and inhomogeneous long T2 signal, which were enhanced with mass or nodosity after contrast administration. The suprasellar region, bilateral cavernous sinuses, and optic chiasma were further invaded. Necrosis, cyst degeneration, and hemorrhage within the tumor could be detected (Fig. 1F–H). A secondary surgery was performed through the left pterional approach. The tumor's texture was firm to elastic and the bleeding was heavy; therefore, we just performed a partial resection, decompressed the optic nerves and chiasm. The sellar MRI after 1 week of the secondary surgery was shown as Fig. 1I to K. Postsurgically, transient central diabetes-insipidus persisted for 2 weeks. Visual field and acuity remained unimproved. Histological evaluation revealed similar morphology and immunohistochemical profiles to the previous specimen. The Ki-67 index for this time increased to 19%. From the perspectives of pathologists, the pathological grade was considered WHO III.

One month after the secondary surgery, the patient came to PUMCH again complaining almost blindness and severe headache. A sellar MRI was arranged, demonstrating that the tumor recurred again to approximately 4.9 × 3.6 × 3.1 cm with necrosis, cyst degeneration, and hemorrhage. The enhancement was inhomogeneous and the surrounding structures and tissues were further invaded (Fig. 1L–P). The third surgery via a transsphenoidal approach was conducted for decompressing. Partial resection was achieved and the visual disturbance and headache were alleviated a lot. Pathological evaluation results were similar to previous, confirming the diagnosis of ASCO. And the Ki-67 proliferation index increased to 45% (WHO III–IV grade, Fig. 2L), highly suggesting its malignancy. The patient has already been followed up for nearly half a year and reported no recurrence of headache and visual deterioration. His right visual acuity was 0.4 and the left was 0.3, evaluated recently.

## 3 Discussion

**Figure 3:**
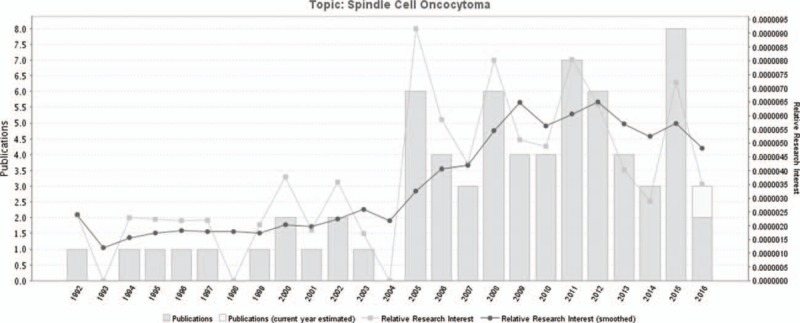
A timeline of the publications related to ASCO. ASCO = adenohypophysis spindle cell oncocytoma.

**Figure 4:**
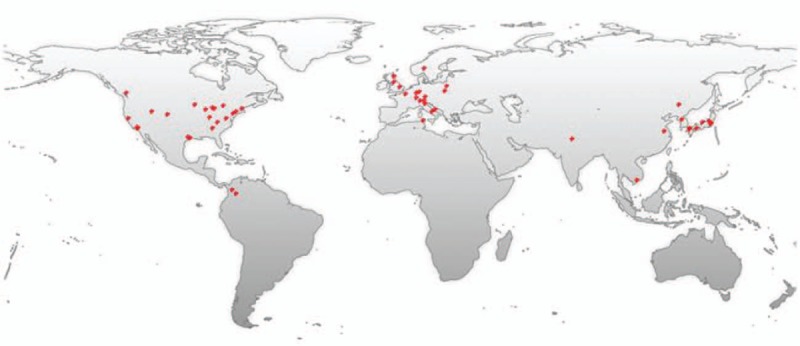
A world map with the global distribution of ASCO-related publications based on the analysis of their geolocational data. ASCO = adenohypophysis spindle cell oncocytoma.

ASCO was first described by Roncaroli et al^[2]^ as a tumor containing fascicles of spindle cells with eosinophilic, granular cytoplasm. They suggested a benign nature based on the absence of cellular anaplasia, mitoses, and necrosis along with a low Ki-67 proliferation labeling index, and that no recurrence was observed in all 5 cases of their series with a mean follow-up of 3 years.^[2]^ ASCO was later identified as a novel entity by the WHO classification of tumors of the central nervous system in 2007.^[1]^ The ASCO-related literature number increased year by year (Fig. 3). The countries that the literatures are from are distributed mainly in East Asia, North America, and Europe (Fig. 4).

**Figure 5:**
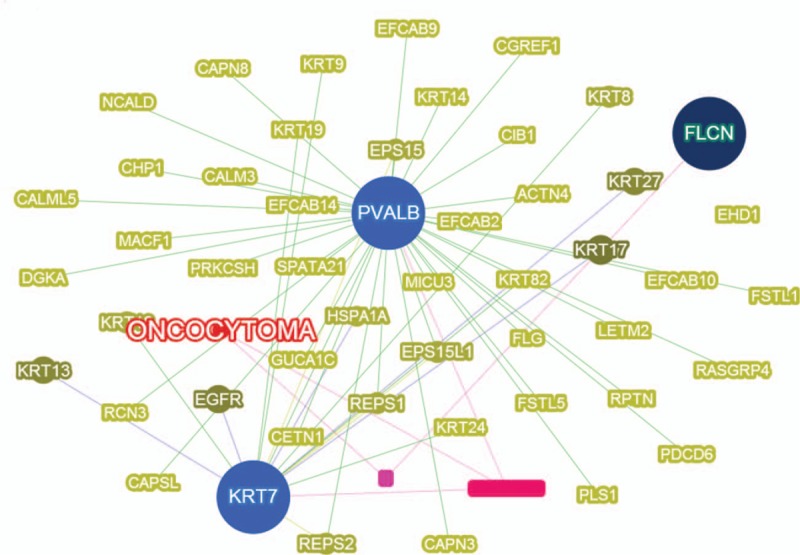
Oncocytoma-related genes by virtue of searching database via Phenolyzer.

ASCO's pathogenesis remains unclear. ASCO was originally thought to originate from folliculostellate cells (sustentacular-cells in the anterior pituitary gland).^[5]^ Nevertheless, TTF-1 was found to be immune-positive in ASCOs, granular cell tumors, pituicytomas, and normal pituicytes, but negative in folliculostellate cells in the anterior pituitary gland.^[6]^ Mete et al^[7]^ believed ASCOs are a variant of pituicytoma due to the same or similar immunohistochemistry profiles and put forward “oncocytic pituicytoma” for these tumors. Ulm et al^[8]^ thought that the similarities observed in many pituitary lesions that include ASCO, pituicytomas, and adenomas, might be due to their same origins in neoplastic transformations of multipotent stem-cells as follicle-stellate-cells with divergent differentiation capacity. However, this is still controversial. Recently, Miller et al^[9]^ found a low mutation-rate and copy neutral profiles of ASCO, consistent with this tumor's low-grade nature. From 1 patient, they revealed a MEN1 frameshift-mutation (p.L117fs) and a cooccurring somatic H-RAS (p.Q61R) activating point-mutation present in primary and recurrent tumors.^[9]^ They also found mutations in FAT1 and SND1, which are closely related to MAPK-pathway activation, suggesting that the MAPK signaling pathway may be a novel treatment target of ASCO.^[9]^ Other oncocytoma-related genes reported before are as seen in Fig. 5.

The ASCO was considered to be a WHO grade I tumor on the basis of benign immunohistochemical characteristics, slow growing nature, and low proliferation index, though tumor recurrences have been mentioned in some literatures.^[10,11]^ Rotman et al^[4]^ described a case with minimal growth over an 8-year follow-up without operation, indicating the options of waiting instead of performing surgery in patients with the risks of operation. Quite different, however, the tumor in our case recurred for several times and the patient underwent 3 surgeries and 1 γ-knife treatment, which was accompanied by a continuously increasing Ki-67 index of 45% finally. It has been beyond the scope of “benign” tumor (WHO I grade) and was deemed as malignant (WHO III–IV grade). To our knowledge, the present case is the first reported one for malignant ASCO.

The radiological features of ASCO are nonspecific and generally could not differentiate these tumors from pituitary adenomas.^[12]^ In MRI, ASCO is often seen as an intra- or suprasellar mass that may have included destruction of the skull base.^[10,13]^ In our case, they consisted of an enhanced mass of the sellar region.

The clinical presentations of patients with ASCO are indistinguishable from those with a nonfunctional pituitary adenoma; this hampers the recognition and diagnosis of ASCO.^[10]^ Generally, the anatomical location determines the clinical features. Those located within the sella turcica typically present with symptoms similar to pituitary adenomas. Lateral expansion into cavernous sinus may compress oculomotor, trochlear, and/or abducent nerves, leading to diplopia and then ocular misalignment. Suprasellar expansion often presents with visual disturbances due to compression of the optic nerve or chiasm; similar to our patient, who developed headaches, and bitemporal hemianopia. When the ASCO compresses the normal pituitary gland, pituitary hormone disorders may follow. In our case, the patient presented diplopia and impaired vision.

Histological examinations are the only ways for diagnoses.^[13,14]^ Typically, this tumor comprises interlacing fascicles of epithelioid to spindled cells with oncocytic-cytoplasm. Mild-to-moderate nuclear-atypia or focal-marked pleomorphism can be observed. The immune-profiles of the tumors are characterized by simultaneous positivity for EMA, vimentin, and S-100 protein.^[15]^ Ultrastructurally, the tumor cells contain abundant mitochondria with lamellar cristae. The neoplastic cells are connected with each other by desmosomes and intermediate junctions.^[16]^

The differential diagnoses for ASCO are wide, with considerations including solitary fibrous tumors, meningioma with oncocytic changes, paragangliomas, pituicytomas, granular cell tumors, null cell adenomas, pituitary adenoma with oncocytic changes, and schwannomas. Of these entities, pituicytomas, granular cell tumors, oncocytic null cell adenomas, and schwannomas represent the greatest diagnostic challenges.^[17]^ Rotman et al^[4]^ have already contrasted ASCO with its main differential diagnoses in detail in their literature.

The main treatment of choice for ASCO is trans-sphenoidal resection plus radiotherapy for recurrent cases. In Dahiya's case, radiotherapy was given soon after a subtotal-resection and no recurrence was observed after a 7-year follow-up period.^[18]^ Contrasting this, a second recurrence was seen in 1 of the cases reported by Kloub et al^[13]^ even after receiving radiotherapy. The prognosis of pituitary tumors is a complex issue in which the recurrence likelihood is only 1 variable.^[19,20]^

## 4 Conclusion

This is the first reported case of malignant ASCO with repeating recurrences and a high Ki-67 index (WHO III–IV grade). This case emphasizes the difficulty in the diagnosis of ASCO as its histological characteristics are very similar to other lesions in sellar-region. A combination of clinical, radiological, immunohistochemical tests is necessary to achieve an exact diagnosis. If there are not any known definitive morphological or other prognosis-predictive factors, a regular and long follow-up plus an aggressive treatment protocol is necessary in ASCO patients.

## 5 Consent

Written, informed consent was obtained from the patient for publication of this case report and accompanying images. A copy of the written consent is available for review by the Editor-in-Chief of this journal.
